# Wildlife–Vehicle Collisions in Tasmania: Tourists’ Attitudes and Behaviour

**DOI:** 10.3390/ani14162413

**Published:** 2024-08-20

**Authors:** Elleke Leurs, James B. Kirkpatrick

**Affiliations:** Department of Geography, Planning, and Social Science, University of Tasmania, Hobart, TAS 7001, Australia; james.kirkpatrick@utas.edu.au

**Keywords:** animal welfare, biodiversity conservation, driver behaviour, roadkill, Tasmania, tourist perspectives, wildlife–vehicle collisions

## Abstract

**Simple Summary:**

Wildlife–vehicle collisions continue to threaten Tasmanian wildlife and cause animal welfare issues. As Tasmanian residents often consider roadkill as part of the landscape, tourists’ attitudes and behaviour could provide new insights when seeking solutions. Using a self-reporting questionnaire, we found that most tourists view wildlife–vehicle collisions as a serious issue, and humans must take responsibility for minimising the problem. Even though residents seem hardened to roadkill, they appear to play an important role in communicating the issue to tourists. Tourists express a willingness to adopt mitigation strategies, such as altering travelling speed or travelling times. However, this research reveals that the importance of safely removing carcasses and checking on the victims and their surviving young is not fully understood. Tourists indicated that they do not know what to do or whom to contact when confronted with injured, surviving, or deceased wildlife. We suggest that the tourism industry, alongside other stakeholders, can play a significant role in raising awareness and education, as well as inform campaigns tailored to Tasmanian residents.

**Abstract:**

The surge in wildlife–vehicle collision research has not yet translated into a substantial decrease in animal fatalities. In line with the prevailing view, we suspect that drivers’ behaviour may be the most crucial element. We address a research gap in drivers’ attitudes towards and behaviour in response to wildlife–vehicle collisions from a tourist perspective. We designed a questionnaire to examine tourists’ attitudes and behaviour in relation to wildlife–vehicle collisions while driving in Tasmania. We found that the respondents’ sociodemographic attributes had minimal effect on their practical responses to roadkill. Tourists consider wildlife–vehicle collisions a serious problem for both biodiversity loss and animal welfare reasons, and their willingness to change their behaviour was high. However, many respondents did not stop to check for surviving pouch young. This inaction resulted either from overlooking the importance of pouch checking or a lack of knowledge on what action needed to be taken. There may also be a lack of understanding that roadkill left on the road leads to secondary roadkill incidents. Even though tourist behaviour does not automatically represent residents’ behaviour, these findings will help to improve and tailor educational approaches to rectify the driver awareness/behaviour gap for both tourists and residents.

## 1. Introduction

Roads traversing or bordering natural habitats frequently disrupt the natural movements of animals, consequently raising the risk of wildlife–vehicle collisions (WVCs). WVCs have devastating consequences for animals, leading to a staggering loss of life, injuries, and orphaned individuals, animal welfare issues, and contributing to the alarming rise in biodiversity decline. Avoidance and mitigation efforts must consider the changeable (e.g., human and animal behaviour) and the unchangeable (e.g., certain landscape features, seasonality). While the term WVC itself focuses on the interaction between wildlife and vehicles, it is crucial to acknowledge that the ultimate responsibility for these collisions lies with the driver [1,2], and behavioural interventions have the potential to reduce WVCs. Besides human errors causing fatalities, familiarisation [3,4] may lead to locals showing indifference, hindering mitigation efforts [5]. Ramp et al. (2016) [3] and Collison (2018) [6] argue that familiarity with the area, wildlife abundance, and past collision experiences influence risk perception and subsequent actions. The research of Ramp et al. (2016) serves as the cornerstone for the present study, offering crucial insights into wildlife protection and responsible driving. The attitudes, perceptions, and actions of tourists may be crucial in motivating mitigation action and are the focus of our research. It is important to note that our purpose is not to pressure tourists to deal with a local issue. However, tourists often feel sadness, anger, and disgust when confronted with roadkill and express feelings of helplessness [7]. Many tourists convey a desire to contribute to a solution.

The significance of the research is that it addresses WVCs with a broader scope than just economic impacts or conservation. Moreover, it draws attention to the human responsibility to consider animal welfare and act accordingly whilst seeking solutions to mitigate the issue. Despite at least an estimated AUD 9.5 million per year in car insurance costs [8], many small animals in Tasmania do not necessarily pose a risk for drivers or their vehicles. In terms of conservation, there is minimal incentive to mitigate roadkill for these species [9]. Government monitoring reveals that frequently road-killed animals maintain healthy, stable, or even increasing populations. Ignoring the fact that the suffering endured knows no species boundaries. Given that animals, just like humans, are sentient, intelligent, and emotional beings, often with strong family structures [10], this moral imperative should be the driving force behind roadkill prevention efforts. Sadly, in our present society, compassion alone is generally insufficient to justify roadkill mitigation strategies, with monetary values being of greater weight. Other key threats causing possible species extinction [11,12,13] also include predation by cats, which cause the deaths of 512 million native animals annually [14], and toxoplasmosis from their faeces can altered wallaby behaviour causing them to become more prone to becoming roadkill [15]. Additional threats encompass dog attacks, agricultural culling (over 850,000 Tasmanian animals in 2021) [16], invasive species, plastic entanglement, poisoning, urban encroachment, and environmental disasters such as bushfires and flooding [17]. Habitat loss further exacerbates these issues, reducing predator evasion, food access, and breeding grounds, while increasing roadkill [2]. This loss also impacts genetic diversity, affecting adaptability to climate and disease [18,19,20]. 

### 1.1. Driver Behaviour

Many road accidents result from human error [21] with drivers’ awareness and behaviour (including reckless driving, distracted driving, and driving under the influence of alcohol and drugs) playing significant roles in WVCs [6,22]. Technology is being developed to prevent and reduce accidents through monitoring systems that detect drivers’ drowsiness and alcohol levels and through collision avoidance sensors that will automatically apply the brakes [23]. Tasmanian research has shown that reducing speed reduces roadkill in the most effective way [24,25,26]. Collison (2019) [6] found that more than 95% of respondents to a questionnaire believe that speed is the sole cause of roadkill. However, Collison (2019) concluded that the driver’s level of observation, rather than speed, is the critical factor in preventing roadkill, a finding confirmed by Yusuf et al. (2023) [23], who also found that the lack of alertness in drivers is the most common cause of accidents. Gupta and Velega (2023) [27] concluded that poor planning, resulting in driving under time constraints, has been found to be the most common reason for animal collisions. Ramp et al. (2016) [3] found a high level of drivers’ awareness regarding wildlife–vehicle collisions and even knowledge of appropriate measures to prevent them. However, there was less adherence to these preventive actions.

Most Tasmanian animals are active between dusk and dawn, when drivers’ visibility is reduced and their attentiveness is lower. Hitting an animal is sometimes unavoidable due to unpredictable movements or other factors like road conditions. Drivers commonly respond to wildlife encounters by resorting to braking and swerving [3,28]. Approximately 50% of Australians have experienced a collision with a kangaroo or other wildlife while driving. Surprisingly, a significant majority (61%) confess they would take risky actions such as swerving or abruptly slamming on the brakes to avoid hitting an animal, as revealed by recent research conducted by Australian Associated Motor Insurers Limited (AAMI) (2023) [29]. One in seven drivers (14%) acknowledges having no clear plan of action in the event of a collision with wildlife, and some even admit they might panic and freeze while behind the wheel. The consequences of road accidents resulting from drivers swerving to evade animals can be severe, leading to outcomes such as fatalities, injuries and harm to both individuals and vehicles. Drivers’ perception of the risk associated with wildlife–vehicle collisions plays a significant role in their behaviour [27]. When collisions rarely result in vehicle damage or injury to the occupant, inducing appropriate behaviour change can be challenging [3].

Solutions that have been suggested include raising awareness and alerting road users to the presence of wildlife through the usage of active/passive signage, as well as influencing driver behaviour and attitude through advertisement campaigns, art, citizen science projects, and social media. Some studies have explored the factors influencing driver behaviour and decision-making when encountering wildlife on roads [3]. Unfortunately, even when awareness and exposure are high, the willingness to make changes in driving behaviour is not high enough [3]. Educational initiatives focusing on traffic play a crucial role in informing and shaping public behaviour and enhancing awareness of traffic risks [30,31,32].

The positive impact of these campaigns is evident in substantial reductions in traffic accidents [33,34] and demonstrate efficacy in mitigating overconfidence among drivers who underestimate traffic risks [35]. Often, several mitigation strategies will be used together. However, depending on several factors (e.g., type of roadside vegetation, volume of traffic, speed of traffic, camber/undulation/windiness of the road), different types of mitigation are more likely to be effective in different situations. In Tasmania, the roads are often winding, sloping, and darkly surfaced, plus fog and rain are expected. The speed limit on rural roads can be as high as 90 km per hour, and on some highways is 110 km per hour. Signage to alert drivers to the danger of roadkill is often in place, but the effectiveness of static signs is debatable [36]. However, a new Tasmanian initiative that used a temporary roadkill memorial campaign followed by wildlife awareness signs, using a wide variety of photos mostly of surviving joeys that were rescued by Tasmanian wildlife carers, appears promising in effectiveness. Community members who want to contribute to roadkill reduction bought signs for their private properties, often with a sign showing the species in that particular spot, to alert drivers to animals present, along with a message to slow down [37].

### 1.2. The Importance of Tourism

The Australian constitution overlooks native animals, entrusting their welfare to a fragmented and inconsistent state and territory regulatory system (Parliament of Australia, 2024). Despite growing societal pressures to address animal welfare concerns from within Tasmanian communities, Australia, and thus Tasmania, remains entrenched in an unjustifiable bias against its native marsupials and monotremes, as noted by Ashby (2022) [38]. This bias underscores the significance of understanding the perspectives of visitors. Research highlights both similarities and disparities in residents’ and tourists’ sentiments toward wildlife conservation [3,39], with cultural background, education, and personal experiences significantly shaping attitudes and behaviour in both cohorts. Residents and tourists often hold divergent views on wildlife conservation, influenced by their respective connections and levels of engagement. Some residents, despite their deep environmental ties, may occasionally overlook wildlife [40] and tourists, with their transient relationships, exhibit a spectrum of attitudes toward conservation.

Nevertheless, a substantial number of tourists strongly resonate with conservation issues. A comprehensive understanding of both groups necessitates context-specific analysis. Tourists make a small contribution to the total of roadkill victims in Tasmania [9]. However, 97% of the tourists encountered roadkill during their visit to Tasmania [7] and experienced the detrimental effects roadside carcasses have on Tasmania’s visitor economy [41].

Since the work of Finn (1978) [42], research has consistently highlighted the unacceptably high incidence of roadkill on Tasmanian roads and its negative impact on the experience of tourists. Although mitigation and educational/awareness efforts (primarily emphasising the need to reduce speed during dawn and dusk) may have made some difference, significant improvements remain elusive. The present research contributes to the further development of mitigation strategies by determining tourists’ attitudes and behaviour regarding roadkill and its mitigation and how this can be translated into a broader perspective. This study is the first to quantify tourists’ experiences, attitudes, and behaviours at a State scale, expanding on previous research focused on local drivers or smaller areas [3].

## 2. Methods

### 2.1. Study Area

Tasmania, nestled off the southern coast of Australia, comprises a diverse range of ecosystems, from alpine areas to ancient rainforests to rugged coastlines. One-fifth of the State is in the Tasmanian Wilderness World Heritage Area. Tasmania has distinctive flora and fauna, including iconic species like the eastern quoll. Several mammal species that are no longer found on the Australian mainland, such as Tasmanian devils, are still found in Tasmania. Tasmania also has the reputation of being the roadkill state of Australia.

### 2.2. Methods

A ten-minute questionnaire with quantitative and qualitative components was used to determine tourists’ experiences, attitudes, and reactions to WVCs and roadkill. Because of COVID-19, the number of respondents was not as high as planned, but it was sufficient to allow confidence in the statistical analysis of the answers to quantitative questions. The qualitative responses captured emotions well beyond the point of saturation [43,44]. To maximise the available resources, both completed and not completed surveys were used. This approach also ensures that all respondents’ insights were included in this research. 

Questionnaires are an essential source of information in tourism research [45]. Social questionnaires commonly assess behaviours and their drivers [46]. Three theoretically informed questionnaires were designed in SurveyMonkey to capture those arriving, those questioned on arrival who were leaving, and other tourists who were leaving (Table 1).

### 2.3. Recruitment of Respondents

Convenience sampling was employed for this study, targeting individuals over 18 years old whose primary purpose of visit was leisure in the State. Those visiting for work or conference purposes were excluded, as the focus was on leisure travellers. Additionally, only independent travellers were included to delve into individual experiences free from external influences. We utilised posters and flyers to recruit respondents, detailing eligibility criteria and providing a web link to access questionnaires. Flyers were distributed at Hobart International Airport, Spirit of Tasmania ferry terminal, campsites across the state, and vehicles with out-of-state licence plates or rental cars. Tourists arriving on the Spirit of Tasmania were also invited through onboard screens for two months. Furthermore, radio and newspaper interviews were conducted to spread awareness about the research and encourage locals to invite visiting friends and family to participate through the research website.

The people who chose to participate and those who did not might have different opinions, which could lead to a bias in the responses given by respondents [47]. Due to the topic’s sensitivity, responder consistency was tested by asking the same question in different formats and as both open-ended and closed questions [48] (for example, questions about human–wildlife relations, such as the need for protection (Arrival question 9, Follow-up question 4, Departure question 10).

**Figure 1:**
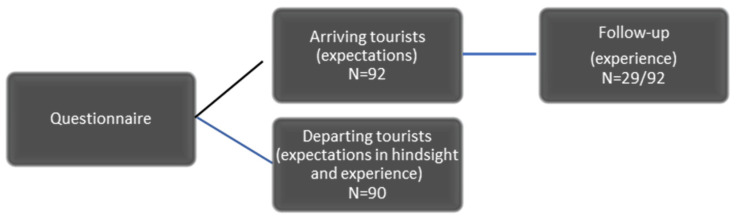
Questionnaire layout of the Bloody Tourism research project. Source: Author.

We approached respondents when they arrived (30 questions) and departed (31 questions). We also approached departing tourists whom we had not interviewed on arrival (47 questions) (Figure 1). The first group, the arriving tourists, were asked to fill out the initial arrival questionnaire (expectations) and then the section on their actual experience upon departure (experience). The other departing tourists were asked the same questions in one questionnaire. 

To understand the effects of personal attributes, we compared age, occupation, gender, and usual residence. A sliding scale ranging from ‘Not important’ to ‘Extremely important’. True/false/I do not know statements were offered, including room for additional comments. To form a broader overall understanding, questions about WVCs in Tasmania included knowledge about the occurrence of animal–vehicle collisions and individual attitudes towards wildlife and animal–vehicle collisions [49,50], plus perceived and actual behaviour towards wildlife [3], and the extent to which animal–vehicle collisions impact the tourist experience [51].

### 2.4. Data Analysis

Minitab version 16 (Minitab Inc., State College, PA, USA, 2017) was used for descriptive and inferential statistics. An Excel database (version 2407) downloaded from SurveyMonkey was curated. Various statistical techniques were employed to analyse our dataset. We utilised the Chi-square test to assess the significance of relationships between categorical predictor variables (such as gender, age, nationality, and visitor type). The significance of variation in practical responses by sociodemographic predictor variables with suitable data was determined using Fisher’s exact probability test except for those related to the date of birth (DOB), for which variation in the DOB was tested against the variation in response using a one-way analysis of variance.

To understand if the importance of wildlife sightings changed with tourists’ experiences, we compared arrival and departure scores using the two-sample *t*-test. Additionally, we applied a one-way analysis of variance (ANOVA) to investigate whether individuals who preferred to be informed about roadkill had stronger feelings than those who preferred not to be informed. One-way ANOVA was also used to explore the significance of other relationships between quantitative response variables and qualitative predictor variables. Finally, Pearson’s product-moment correlation coefficient was employed to assess the significance of linear relationships between continuous variables. These statistical methods facilitated a comprehensive data analysis, enabling us to derive meaningful insights and conclusions regarding the relationships and differences within the dataset. We note that these statistical techniques adjust their assessment of the probability of a relationship or difference occurring by chance by the number of individuals that are used in the analysis, so small sample numbers only become a problem when numbers are very low, as in Chi-square tests with expected values less than 5. We did not use the tests in this sort of situation. On occasion, it was not relevant to distinguish between arrival/follow-up or departure datasets, and therefore, a combination of these datasets was used.

## 3. Results

### 3.1. Respondents

Overall, 92 arrival questionnaires, 29 follow-up questionnaires and 90 departure questionnaires were collected. Of the respondents questioned upon departure, 64% were first-time visitors, and 36% were repeat visitors. For the arriving group, first-time visitors (48%) and repeat visitors (52%) were almost identical in number. More Australians (69%) than overseas visitors (33%) participated in the arrival survey. The departure group was almost identical in Australians (48%) and overseas (52%).

### 3.2. Mode of Transport

As the airport was the primary place for respondent recruitment, car rentals were used the most for getting around (66.5%) by all respondents. Others used their own car (14.5%), a motorcycle (1.3%), a campervan/motorhome (10.5%), a bicycle (2%), and public transport (5.5%). A few borrowed vehicles from friends and family or had a private driver. Respondents were either the primary driver or not driving (40%), and 20% shared the driving equally. The modal length of stay was 1–7 days (79%). 

### 3.3. Wildlife and Roadkill Perspectives and Values

Our survey data showed that seeing wildlife during the holiday was rated as very important. On a scale of 1 (not important) to 10 (extremely important), tourists ranked seeing wildlife at an average of eight. The perception of individuals of the importance of seeing wildlife did not change between arrival and departure (*t* = −1.3, DF = 144, *p* = 0.196). Their feelings on the seriousness of the issue of roadkill also averaged a score of eight with no variation between arrival and departure (t = 0.37, DF = 144, *p* = 0.714). Most (90%, *n* = 126) of the respondents know that wildlife–vehicle collisions occur primarily between dusk and dawn. The remaining 10% *(n* = 15) indicated they did not know if this was the case. Very similar results were found when asked if it was true or false that many Australian mammals are ‘marsupials’, which means that their babies are carried in the mother’s pouch (true 95%, *n* = 139, false 1% (*n* = 2), and 4% *(n* = 5).

**Figure 2:**
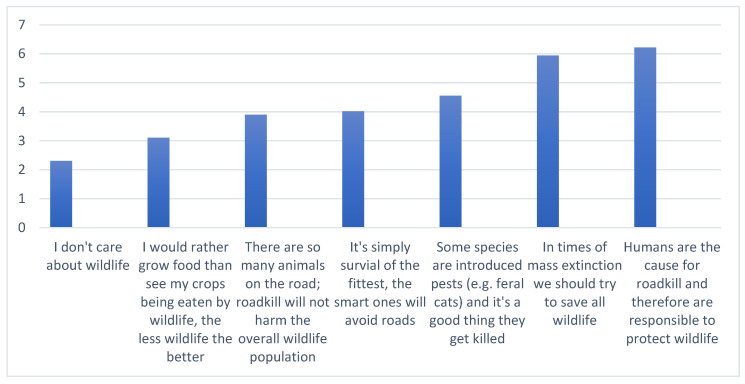
Mean responses on a seven-point Likert scale for statements in the questionnaire about wildlife and roadkill. 1 = strong disagreement; 4 = neutral; 7 = strong agreement.

To elicit a more specific response, the broad term ‘wild animals’ was employed to gauge how tourists view animals in Tasmania. No respondents selected the option to label ‘wild animals’ as ‘mostly pests’ or ‘pests to a certain degree’. Nevertheless, comments revealed that individuals recognised a distinction among native animals, with some asserting that feral cats and rabbits did not warrant protection. Threats to populations and the diversity of animals were also recognised in comments. Three respondents felt indifferent about wildlife protection and considered animal extinction as an acceptable process. The majority acknowledged that humans are the reason for roadkill and should take responsibility (Figure 2).

**Figure 3:**
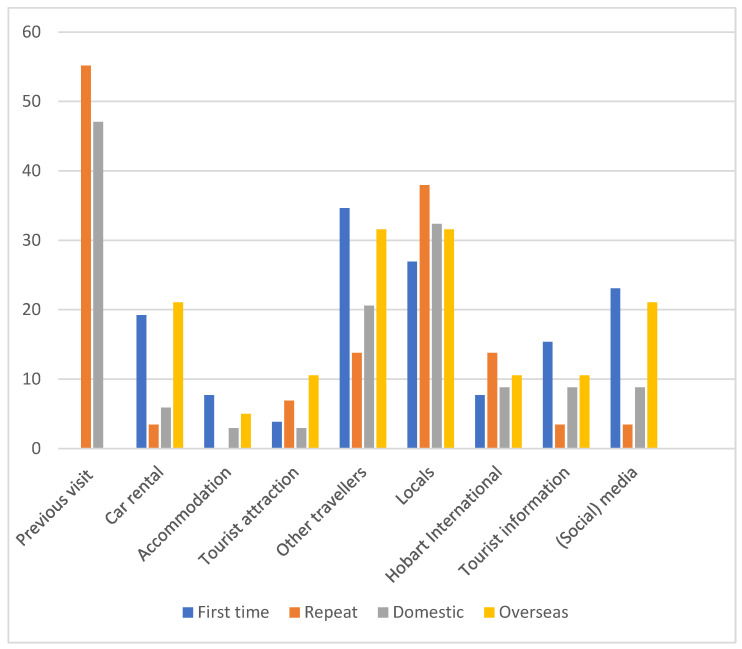
Percentages of first-time and repeat respondents, and domestic and overseas respondents, who gained information on roadkill from various sources named on the questionnaire.

Most respondents (64%, *n* = 89) heard about the occurrence of wildlife–vehicle collisions (roadkill) in Tasmania before their visit (Figure 3). However, many respondents mentioned they were not aware of the extent and the amount the encountered. The most mentioned sources for knowledge of wildlife–vehicle collisions in Tasmania were ‘from a previous visit’ (37%, *n* = 31), ‘from locals’ (36%, *n* = 23), and ‘other travellers’ (28%, *n* = 19). Other options in this question were ‘social media’, a car rental company’, ‘the accommodation’, ‘the tourist information centre’, and ‘Hobart International Airport’. Some mentioned that the prevalence of roadkill in Tasmania is common knowledge and part of travelling in Australia. 

Tourists who were unaware of roadkill would have preferred to be informed about the elevated levels of roadkill in Tasmania. (42%, *n* = 59). A minority would have preferred not to be told (16%, *n* = 22), and a few were unsure about this (12%, *n* = 17). The remainder indicated they were already aware (29%, *n* = 41).

### 3.4. Roadkill Impact on Travel Planning

The concern about wildlife on the road impacted the travel plans of 54% (*n* = 38) of the arriving tourists. Contrasting departing respondents, of whom only 28.79% *n* = 19) indicated that their plans were impacted and 71% (*n* = 47) said it did not. The answers of the arriving tourists (what they expect to do) were consistent when asked the same question on the follow-up survey (what they did). 

**Figure 4:**
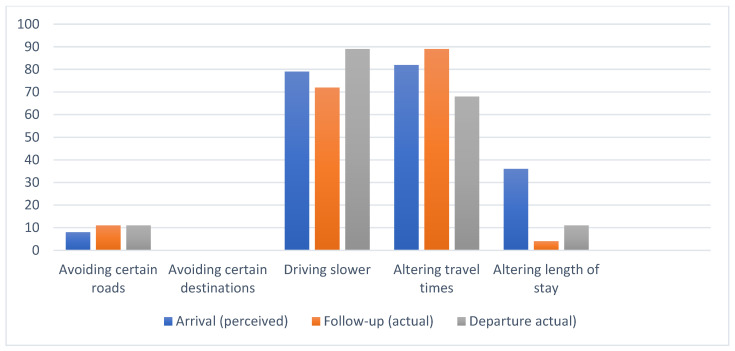
Percentages of participants in each of the arrival, follow-up, and departure groups who, after indicating concern about roadkill, believed that they would adopt, or did adopt, various strategies named in the questionnaire to mitigate roadkill.

The tourists who indicated that wildlife encounters would or did impact their travel plans (arrival/departure combined) drove slower (82%, *n* = 48), altered travel times on day trips, for example, to avoid driving at dusk and dawn/night (79%, *n* = 45) and to a lesser extent avoided certain roads (11%, *n* = 5) or altered their length of stay (23%, *n* = 16). The results for perceived and actual behaviour are shown in Figure 4.

### 3.5. Experiences of Wildlife–Vehicle Collisions

Of the 92 respondents who answered the question of whether they had seen wildlife on or near the road, 63% (*n* = 56) had seen living wildlife, and 97% (*n* = 90) had seen deceased wildlife. Four respondents (6%) encountered injured wildlife. During their visit, 36% (*n* = 32) of the respondents experienced near misses (close calls) with animals with their vehicle. During their visit, 21.4% (*n* = 15) experienced one or more collisions with wildlife with their vehicle, primarily birds. None of the respondents claimed to have damage to their vehicle or reported human injuries due to their WVC experience. 

### 3.6. Predicted and Actual Behaviour in Relation to Wildlife–Vehicle Collisions

**Figure 5:**
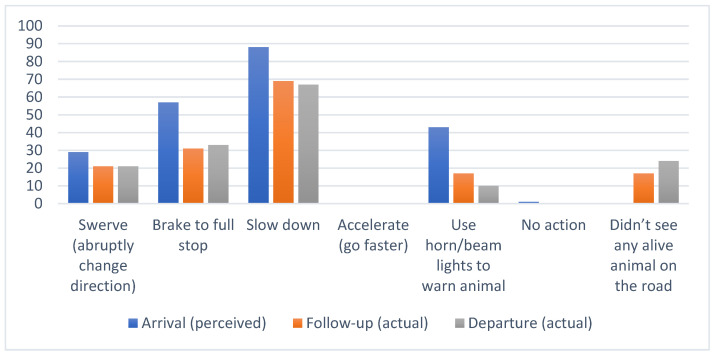
Percentages of respondents who stated that they did not stop when they saw injured or dead animals on the road and who gave particular reasons for not stopping.

Respondents were asked to predict what they thought they would do when, as drivers, they saw an animal on the road (Figure 5). Some added comments that this would depend on the road, traffic conditions, or personal safety. Eighty-eight percent predicted they would slow down, but in reality, this was 68%. Arriving respondents also expected to use the brakes to a full stop, but only 32% did. The most significant difference in perceived vs. actual behaviour is the use of the horn as a preventive action. 

From the 63 departing respondents who responded to whether they stopped for an injured or dead animal, the vast majority responded negatively (81%, *n* = 48). The remaining respondents said they sometimes stopped. Of the 29 follow-up respondents, 90% did not stop, only one stopped (3%), and two said they stopped sometimes (7%). Most said they would stop for injured wildlife but not dead animals, one reason being the amount of roadkill and the state of the carcass, e.g., days old and bloated or exposed guts. A respondent demonstrated that they knew the need for pouch checking, but no one mentioned the possibility of secondary roadkill in this context.

**Figure 6:**
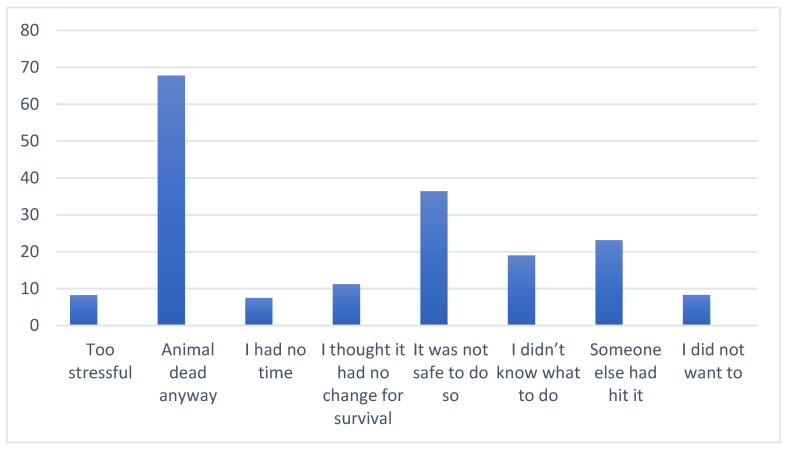
Percentage of respondents who gave reasons for not stopping.

The vast majority said that they did not stop because the animal was dead anyway (Figure 6). Other reasons cited by more than 10% of respondents were ‘it was not safe to do so’, ‘someone else had hit it’, ‘I didn’t know what to do’, ‘I thought it had no chance for survival’.

Respondents who stopped when confronted with the results of a WVC, either by them or by someone else, dead or alive, did so because it was safe to do so (29%, *n* = 16) because they considered the possible surviving joey in the pouch (29%, *n* = 16), or they thought the animal had a chance of survival (21%, *n* = 13). Only eight (22%) did so because the animal was still visible from the road. Only two respondents contacted an animal rescue service. The vast majority (92%, *n* = 78) did not. Eight respondents mentioned they did not know whom to contact. In the open comment section, people wrote that contact details are rarely shown on the roads and suggested that visible wildlife contact details would be helpful.

Roadkill can be reported via a roadkill app. Over half of the respondents (52%, *n* = 45) said they did not use the app. There was a difference between departure (52%, *n* = 31) and follow-up (38%, *n* = 11) respondents who answered they were unaware of such an app. It must be noted that the follow-up respondents were told about the app when they signed up at the time of participation, as the app was offered as an option to record emotions around the event of WVCs.

Arriving respondents thought they would be more likely to stop if they hit an animal than if the animal had been hit by another vehicle, were almost equally divided between the perceived relevance of stopping for roadkill if they hit it (48%, *n* = 33) or someone else’s (52%, *n* = 36). Respondents expected to be more upset if they hit it themselves and would feel a greater responsibility. When departing respondents were asked what prevented them from stopping, 21 (*n* = 13) mentioned that someone else had hit it, with only a few respondents actually hitting an animal, and 5 mentioned they stopped because they hit it (8%). One respondent commented that moving roadkill off the road is a form of showing it died in dignity. When not responsible, another respondent mentioned how they always apologise to the deceased animal for others’ actions.

### 3.7. Mitigation Willingness

The sociodemographic attributes of the respondents had minimal effect on their practical responses to roadkill (Table S1). There was no variation in anticipated and actual responses or reasons for stopping (Table S1). People from overseas are more likely to slow down (78%) than Australians (55%). Of the reasons for not stopping, only one out of five had significant differentiation by sociodemographic variables. Females (27%) were more likely than males (7%) to say they did not stop because they did not know what to do (Chi2 = 4.19, DF = 1, *p* = 0.041). Younger people were more likely than older people to say the same thing (ANOVA, F = 5.26, DF = 1, 76, *p* = 0.025).

Some respondents believe there is much humans can do to mitigate roadkill:

“*I bet the death of these animals could have been avoided if road users were better prepared and made aware of their presence*”.

Departure, no 5

“*They are innocent creatures who could be saved with the introduction of some awareness measures and reduction in speed*.”

Departure, no 51

Secondary roadkill was also mentioned:

“*Sad to see so many dead animals on the road; it encourages scavengers, which in turn can be hit by vehicles*”.

Departure, no 44

“*I feel disgust and anger at the indifference of humans who do not stop and help injured wildlife, or remove it off the road to prevent carrion feeders from being killed when eating the carcass*”

Departure, no 17

One tourist suggested:

“*I hope you can change asap the current sad situation and change the minds of the locals, and inform the tourists before arriving how to behave. When we rented the camper, we asked what we should do when we hit an animal. The answer: nothing, we have so much of it will not matter you only to call someone if something is with your car. This was a very bad statement.*”

Departure, no. 1

Other suggestions made by tourists include calling for more driver awareness, speed cameras in roadkill hotspots, more signage, including wildlife crossing signage, and all rental cars to have roadkill information sheets, including contact details for wildlife rehabilitation centres (Hobart International Airport has partly implemented this). Another respondent mentioned that Tasmania is a poor state and does not have the funds to invest in roadkill mitigation projects similar to those on the mainland. Respondents mentioned that local attitudes make a significant contribution to the roadkill numbers.

## 4. Discussion

Understanding tourists’ wildlife perception and knowledge can uncover gaps or misconceptions. Ultimately, this understanding contributes to a deeper understanding of global human–wildlife coexistence and facilitates behaviour alignment with conservation goals.

We recognise some limitations of our work. The sample size may impact the validity and the generalisability of the findings. Also, the use of the Tasmanian devil as a promotional design may have influenced the answers of the respondents and their willingness to participate in a wildlife-related survey. The follow-up survey was likely to be accepted by those feeling strongly about the topic, whereas the departure survey is likely to have captured a wider range of tourists. Self-selection of participants always requires caution in interpretation. Agreement to answer the questionnaire may have reflected an interest in animal welfare, which, in turn, may have been responsible for the high ratings on the seriousness of the issue [52]. Although the present study is valid for those who answered the questionnaire, we cannot generalise to all self-drive tourists. 

Respondents acknowledged the current pressure on biodiversity and considered it a human responsibility to look after and protect our wildlife. However, despite the knowledge that most road-killed animals are marsupials in Tasmania, our research confirmed the existing misconception that there is no need to stop for roadkill. Female and younger respondents indicated they did not stop for roadkill because they had no idea what to do; this finding suggests a targeted campaign to reach these groups. This knowledge gap appears to be also common among residents, as the question of what to do with an injured animal is a prominent question on local social media platforms such as Facebook. The majority did not stop because “the animal was dead anyway”. When safe to do so, checking animals that have been hit by a vehicle is essential from an animal welfare perspective. Many respondents demonstrate an awareness that a substantial proportion of road-killed Tasmanian wildlife is marsupials, known for carrying their young in pouches. Despite having this knowledge, respondents did not engage in pouch checking to look for potential survivors, confirming a knowledge gap regarding the significance of checking for joeys in the pouch or knowing what appropriate actions to take.

Our findings provide strong evidence that awareness about secondary roadkill needs to be improved. Pulling carcasses off the road can also significantly reduce the secondary roadkill of scavengers. Grieving partners or offspring are also often found next to the carcass. In addition to the importance of removing a carcass from the road, people need to be educated on where to leave it to avoid birds of prey getting trapped in fences or colliding with powerlines (Bunker, ecologist, 2023, pers. comm.).

A Canadian study by Vanlaar et al. (2018) [53] concluded that knowledge only sometimes translates into appropriate behaviour. They found that around 40% of drivers need to learn the safest method to prevent WVCs. Our research findings align with such existing studies, indicating that drivers often possess the knowledge and intention to engage in safe behaviours, such as slowing down and maintaining a straight course when confronted with an animal directly in their path, but do not necessarily do so when confronted with the reality. However, despite having this knowledge and intention, drivers do not consistently adopt these safe behaviours [3]. Interestingly, respondents in this research appeared to slow down rather than swerve.

Research aimed at comprehending behaviour is crucial, given the complexities surrounding roadkill issues. Often, the approach is overly simplistic, with many expecting a one-size-fits-all solution. While wildlife behaviour can be influenced to some extent, as seen in roadkill crossings with features like rope bridges, technological advancements, particularly in AI programmes for heat detection in cars, show promise in reducing collisions. Nevertheless, the most impactful solution remains the alteration of human behaviour.

We found that locals played a pivotal role in roadkill awareness. Most respondents learned about roadkill through interactions with residents. Crafting effective solutions necessitates a targeted approach, emphasising messages that locals can readily convey to tourists and fostering meaningful conversations that are already underway.

The tourism industry can play a vital role in heightening awareness and engagement for tourists while alleviating feelings of helplessness. For instance, to promote responsible actions like stopping for roadkill when safe, rental cars in Tasmania could come equipped with a basic rescue kit (e.g., gloves, hi-vis vest, manufactured pouch), along with first responder information and contact details for Bonorong, Tasmania’s wildlife rescue network. On a local scale, such an initiative would necessitate the expansion of rescue networks and an increase in the number of wildlife carers, addressing the current shortage of registered carers. Although the broader issues are beyond the scope of this research, a comprehensive review of roadkill rescue, considering the mental, physical, and financial welfare of Australian wildlife carers [25], provides valuable insights. Additionally, tourist information points could be supplied with similar rescue kits. Notably, there should be no pressure on tourists but rather an available resource for those seeking guidance on what to do.

The government can contribute by implementing a thoughtfully crafted governmental Roadkill Mitigation Strategy, as this is imperative for addressing the persistent threats posed to wildlife and human health due to WVCs. Ideally, this should be adapted to local councils to address area-specific requirements. Within these strategies, local wildlife groups need to be supported in attempts to encourage resident engagement. However, in dealing with government bureaucracy, it appears strategy plans are the highest-level documents outlining missions and values; sometimes, an action plan can achieve more in a shorter time.

## 5. Conclusions

Seeing wildlife was considered by respondents as an important part of their trip. The arriving respondents who were worried about wildlife on the road expected to alter their travel times or drive slower and acted upon this expectation. The departing respondents said they slowed down more than they altered travel times. Just over half the respondents were aware of the likelihood of seeing roadkill; in reality, 97% did. Respondents believe that in the context of current accelerating biodiversity loss, roadkill is a serious issue caused by humans. Respondents who were not aware of the roadkill issue indicated they preferred to be told upfront and expressed a need for more details on what to do and who to contact. Many tourists learned about the roadkill issues from Tasmanian residents.

This research highlights the importance of the assumption that no action is needed when the animal is dead anyway, which needs to be addressed. It not only dismisses the value of the individual animal, but it also indicates a lack of understanding of the importance of avoiding secondary roadkill. The tourism industry can play a significant role in raising awareness and education to meet tourists’ expectations, as well as Tasmanian residents. Our research contributes to a further understanding of driver experiences, attitudes, and behaviour and highlights the importance of awareness campaigns for both tourists and Tasmanian residents. 

## Figures and Tables

**Table 1:** Questionnaire section with questions relating to wildlife–vehicle collisions.

	Questions Relating to Wildlife–Vehicle Collisions (Roadkill) on Tasmanian Roads
1	Have you heard about the occurrence of wildlife–vehicle collisions (roadkill) in Tasmania?
2	Where did you hear about the occurrence of wildlife–vehicle collisions in Tasmania? Please select all that apply (if you answered yes to question 1).
3	Has concern about wildlife on the road impacted your travel plans? (Yes/No)
4	If yes, how did concern about wildlife on the road impact your decision-making? Please select all that apply.
5	Would you prefer to have been informed about the elevated levels of roadkill in Tasmania before your visit?
6	Using the slider, please rate on a scale of 1 (not serious at all) to 10 (very serious). Please tell us how you feel about the issue of roadkill.
7	If you were the driver and you saw an animal on the road, what do you predict you would do? Please select all that apply.
7a	In most cases, if you saw an animal on the road (alive), what did you do? Please select all that apply.
7b	If you took avoidance actions, what did this depend on? (open question)
8a	What is your likely response when confronted with injured wildlife on the roads?
	Please answer if you stopped driving/riding when confronted with injured or dead wildlife on the road. (Y/N/Sometimes)
8b	What prevented you from stopping when confronted with a wildlife–vehicle collision? Please select all that apply.
8c	When you were confronted with the results of a wildlife–vehicle collision, what caused you to stop? Please select all that apply.
8d	Have you contacted an animal rescue service? If yes, who did you contact?
8e	Have you used a roadkill app to report any roadkill you have encountered?
9	What is your likely response when confronted with dead wildlife on the roads?
10	Will your response depend on if you hit it or if someone else did?(For departure integrated in Q28 and Q29 of the departure questionnaire)
11	On a scale of 1-5, how willing are you to take the following actions to minimise roadkill?
12	Have you encountered wildlife (dead/injured/alive) on/near the roads during your travels in Tasmania?
13	Please indicate what wildlife you have seen (choice between living, injured, dead).
14	On a scale of 1 (not at all) to 10 (very much), please rate how the presence of deceased or injured wildlife on the roads impacted your tourist experience.
15	During this visit, have you experienced near misses (close calls) with animals with your vehicle? If yes, how many?
16	During this visit, have you experienced collisions with animals in your vehicle? If yes, how many?
17	Have you sustained damage in a (near) collision with an animal?
18	Please indicate the level of damage.
19	Did you claim insurance?

## Data Availability

The dataset is available on request from the authors.

## Supplementary Materials

The following supporting information can be downloaded at: https://www.mdpi.com/article/10.3390/ani14162413/s1, Table S1: Practical responses to roadkill by sociodemographic variables. Fishers exact probability test was used on all relationships except those related to date of birth (DOB) for which variation in DOB was tested against the variation in response using one way analysis of variance. The figures for all except DOB are the percentage of the number in the class of the predictor variable. For DOB the figures are the mean year of birth. Bold pairs with an asterisk are significantly different at *p* < 0.05.
