# Supplementary material for: Wildlife–Vehicle Collisions in Tasmania: Tourists’ Attitudes and Behaviour
Source: Animals (Basel). 2024 Aug 20;14(16):2413. doi: 10.3390/ani14162413 (PMC11350784; doi:10.3390/ani14162413)
Supplement: Supplementary file 1 [file animals-14-02413-s001.zip › animals-3116471-supplementary.pdf]

Supplementary Material

Table S1. Practical responses to roadkill by sociodemographic variables. Fishers exact probability test was used on all relationships except those related to date of birth (DOB) for which variation in DOB was tested against the variation in response using one way analysis of variance. The figures for all except DOB are the percentage of the number in the class of the predictor variable. For DOB the figures are the mean year of birth. Bold pairs with an asterisk are significantly different at $p < 0.05$

Response	Gender		Origin		DOB		Professional		Retired	
	M (%)	F (%)	Aus	OS	Yes	No	Yes	No	Yes	No
Anticipated response										
Drive more slowly	85	95	96	89	1977	1988	92	93	100	91
Impact plans	36	45	42	42	1977	1972	39	44	33	43
Actual response										
Avoid bad roads	7	5	7	4	1978	1974	8	3	0	6
Drove more slowly	32	36	36	35	1977	1973	37	30	18	36
Altered travel times	32	35	36	33	1977	1973	32	31	23	33
Altered stay	11	12	9	16	1978	1974	16	8	5	12
Experience with wildlife										
Saw living wildlife	60	60	53	71	1976	1977	**70**	**50***	33	62
Near misses	45	33	35	39	1980	1974	42	31	22	38
Swerved	27	16	16	22	1972	1978	13	21	20	17
Braked hard	27	30	29	32	1978	1976	31	26	30	28
Slowed	60	66	**55**	**78***	1976	1978	64	56	50	61

Horn/high beam	10	13	14	10	1979	1976	11	11	10	11
Stopped	10	16	13	20	1975	1976	19	15	11	18
Why not stop?										
Animal dead	62	71	64	74	1977	1978	73	59	87	63
No chance of surviving	12	10	9	11	1971	1978	16	4	13	9
Not safe	32	31	30	35	1980	1975	32	30	25	32
Not know what to do	**7**	**27***	18	23	**1984**	**1974***	13	24	13	20
Hit by others	15	25	25	17	1978	1976	21	22	12	22
Why did you stop?										
It was visible	33	22	43	13	1972	1976	33	14	0	27
Had a chance	0	33	50	22	1983	1972	25	37	0	33
Possible pouch young	17	42	67	44	1976	1975	62	38	100	47
Safe to do so	18	27	67	38	1974	1982	57	37	100	43
Knew who to contact	25	42	50	27	1981	1975	57	38	0	37

Methods

The significance of variation in practical responses by sociodemographic predictor variables with suitable data (Table S1) was determined using Fishers exact probability test except for those related to date of birth (DOB) for which variation in DOB was tested against the variation in response using one-way analysis of variance. All analyses were undertaken in Minitab16.

Results

The sociodemographic attributes of the respondents had very little effect on their practical responses to roadkill (Table S1). There was no variation in anticipated and actual responses or reasons for stopping (Table S1). Out of seven responses under the heading of experience with wildlife, only slowing in relation to animals on the road varied significantly, with people from overseas more likely to do so (78 %) than Australians (55 %). Out of the reasons for not stopping, only one out of five had significant differentiation by sociodemographic variables. Females (27 %) were more likely than males (7 %) to say that they did not stop because they did not know what to do. Younger people were more likely than older people to say the same thing (ANOVA, $F = 5.26_{1, 79}$, $P = 0.025$).
